# Supplementary material for: Are carbon emissions trading and green financial instruments synergistic? -Comprehensive quantitative research based on content analysis
Source: PLoS One. 2024 Mar 7;19(3):e0298601. doi: 10.1371/journal.pone.0298601 (PMC10919601; doi:10.1371/journal.pone.0298601)
Supplement: S1 Data — (ZIP) [file pone.0298601.s001.zip › Data/地级市Z_F工作报告.zip!/╣π╢1⁄2/╜╥╤⌠╩╨/└┤╫╘╣1⁄2╓┌║┼í╛┬φ┐╦╩2╛▌═°í┐_2003.docx]

各位代表：

我代表市人民政府向大会作政府工作报告，请予审议，并请政协委员和列席人员提出意见。

过去五年工作回顾

市第二届政府任期的五年，是我市经受严峻挑战和考验，克服困难，稳步发展的五年。五年来，市政府在省委、省政府和中共揭阳市委的正确领导下，在市人民代表大会及其常委会的监督下和市政协的支持下，高举邓小平理论伟大旗帜，全面贯彻“三个代表”重要思想，坚持以经济建设为中心，以加快发展为主题，以结构调整为主线，积极实施“工业立市、科教兴市、外向带动、可持续发展”四大战略，按照“稳中求快，强化管理”的工作方针，抓住机遇，团结拼搏，攻坚克难，开拓进取，全市国民经济持续健康发展，社会各项事业全面进步，人民生活水平不断改善，基本完成了市二届人大一次会议确定的各项任务。

一、国民经济持续较快发展

2002年，全市国内生产总值442.09亿元，比上年增长8.3%，比1997年增长67%，五年年均增长（下同）10.8%。地方财政一般预算收入9.91亿元，比上年增长7.97%，按可比口径计算，年均增长8.04%。工业发展步伐加快。纺织服装、化学工业、五金机械、食品饮料和电子信息等支柱产业不断壮大，工业园区建设水平不断提高，地方特色经济迅速发展。品牌战略取得成效，全市著名商标从1997年的1件增至2002年的14件，知名商标从零增至82件，4家企业产品获名牌产品称号，榕江牌酱油获国家免检产品证书。蓬勃发展的民营经济成为我市经济建设的主力军和生力军，2002年，全市民营工业产值占工业总产值73.5%，省级民营科技企业75家。榕泰、康美、雷伊3家民营企业先后成功上市。2002年，全市工业总产值786.84亿元，比上年增长11.5%，年均增长12%。农业和农村经济稳步发展。粮食生产连年获得好收成。农业产业化经营取得成效，特色农业快速发展，6大农业支柱产业和12个农业商品基地建设水平进一步提高，15家省、市级农业龙头企业和1800多家农产品加工企业带动作用更加明显，农业市场准入体系和标准化体系建设步伐加快。2002年，全市农业总产值71.32亿元，比上年增长5%，年均增长6.3%。以商贸流通业为基础的第三产业加速发展。2002年，全市第三产业增加值136.62亿元，比上年增长8.6%，年均增长9.6%。经济结构进一步优化，三次产业构成比例由1997年的20.8∶52.0∶27.2转变为2002年的18.8∶50.3∶30.9。

二、基础设施建设取得突破性进展

五年来，全市全社会固定资产投资总额344.44亿元，年均增长7.7%。建成了遍布全市的公路网，公路密度达到每百平方公里77.7公里，普惠高速公路、省道236复线等一批公路建成通车，全面实现了行政村村村通公路。潮汕民用机场获国家批准立项。新建和扩建3座220千伏输变电站、7座110千伏输变电站，惠来海湾石风电场建成投产，农网第一期改造工程全面完成。2002年，全市固定电话交换机总容量93.4万门，平均每百人拥有固定电话13.1部、移动电话13.7部。农村改水工程步伐加快，榕城围北堤、横江水库和揭东炮台、地都、惠来东陇海堤等一批水利工程进一步除险加固，省人大关于解决小型水库安全隐患问题议案得到办理落实。

城乡建设取得新成绩，全市96%的镇完成规划编制，16个镇列入省中心镇规划建设行列，市区“两河四岸”规划逐步实施。市政配套设施进一步完善，市港务局新码头、中央储备粮揭阳直属库、岐山汽车站、进贤商业步行街等一批项目竣工投入使用，榕东、梅东、环市北河大桥建成通车。市容市貌明显改观，市区主要出入口、“一道两路”、引榕干渠等得到整治。市区建成区面积扩展至27.6平方公里。揭阳作为一个新兴中等城市已初具规模。

三、市场开拓成效显著

采取“政府搭台，企业唱戏”等形式，先后在乌鲁木齐、太原、西安和南宁等地开展商品展销活动，举办了首届中华阳美（国际）玉器节、普宁国际衬衣暨纺织服装交易会、普宁中药材交易会，特别是揭阳市2002年经贸洽谈暨建市10周年纪念活动，进一步敦睦了乡谊，凝聚了人心，活跃了经贸。市场建设得到加强。2002年，全市有各类市场156个，其中有16个市场年贸易成交额１亿元以上，全市社会消费品零售总额148.65亿元、集贸成交额232.1亿元，年均分别增长12.4%和8.3%。国际市场进一步拓展。坚持“走出去”与“引进来”相结合，积极组织企业参加广交会和省在境外举办的经贸活动，引导和支持企业参加各类国际博览会，外贸出口和利用外资出现恢复性增长。2002年，全市外贸出口4.43亿美元、实际利用外资2.02亿美元，分别比上年增长48.9%和30.8%。

四、各项改革取得重要成果

政府机构改革顺利完成，市级政府机构、行政编制分别精简27.3%和35.5%，县（市、区）、乡（镇）机构和人员也得到较大幅度的精简。政府职能进一步转变，行政审批制度改革稳步进行，两轮改革共取消和调整各类审批事项565项，占总数的34.8%。国有企业改革继续深化，国有及国有控股工业企业改革和脱困“三年两大目标”基本实现，国有资产监管和运营体系逐步建立。公共财政体制框架初步形成，部门预算试点、政府采购等工作有效推进。产权、技术和房地产等要素市场加快发展，市场体系不断完善。农村第二轮土地承包工作顺利完成。农电“两改一同价”取得较大进展，农村住宅到户电价降至0.79元／千瓦时以下。城市管理相对集中行政处罚权试点工作稳步推行。住房制度、城镇户籍制度改革和工商、医药、卫生、粮食流通、质量技术监督等管理体制改革顺利进行。

五、发展环境明显改善

以加强信用建设为核心，深入开展打击逃税骗税、逃废债务、制假贩假、走私贩私、吸毒贩毒等违法犯罪活动，特别是打击骗取出口退税斗争取得重大成果，非法传销、卷烟制假、无证生产螺纹钢等问题得到有效整治。金融监管力度加大，依法清收城信社和农金会资产进展顺利。建筑、土地、文化、旅游、中介服务等市场秩序进一步规范。安全生产责任制和消防安全责任制进一步落实，事故隐患明显减少。严打整治斗争取得重要成果，重点地区和突出问题得到集中整治，各种刑事犯罪和社会丑恶现象受到有力打击。严密防范和坚决打击了“法轮功”邪教组织破坏活动。社会治安明显好转，我市成为全省刑事犯罪发案率较低的地区之一。

计划生育工作扭转了被动落后局面，稳步走上依法管理的轨道。1999年以来连年顺利通过了省的人口与计划生育目标管理责任制考核，计划生育村民自治先行点经验在全省推广，得到省委省政府的充分肯定。殡葬改革取得突破性进展，2001年以来连续二年被省政府通报表彰。“一控双达标”工作取得成效，榕江、练江、枫江水污染问题得到初步整治，环境保护目标责任制考核连续三年被省评为优秀。土地资源保护力度不断加大。黄岐山等三个省、市级森林公园建设初具规模，全市森林覆盖率达51.6%。

六、社会各项事业全面进步

坚持用邓小平理论、“三个代表”重要思想武装干部群众，党的十六大精神宣传教育活动深入开展。“五个一工程”再创佳绩，“文化艺术上山下乡工程”五年工作目标顺利实现。群众性精神文明创建活动扎实有效。公民道德建设继续加强，“致富思源，富而思进”、“崇尚科学、反对邪教”、“治乱从城镇开始”、“告别陋习，倡导文明行为”等活动富有成效，“聚三江人心，创时代伟业”的揭阳精神深入人心，全市人民的责任感和凝聚力增强。社会科学、新闻出版、广播电视、文史档案等事业不断进步。

教育基础设施不断改善，普及九年义务教育得到落实，改造薄弱学校取得成效，素质教育全面推进。揭阳职业技术学院、潮汕职业技术学院、市综合中专、科技中专、体育运动学校等大中专院校相继建成，教育体系日趋完善。科技成果向现实生产力转化步伐加快，科技对工业经济增长的贡献率达45.6%。五年来，全市高新技术企业由８家增至17家，实施国家和省各类科技计划141项。科技兴农力度加大，全市种植业、畜禽业良种率分别达到90%和80%以上。政府信息平台和企业信用信息网建成开通。科普工作得到加强。

卫生工作取得新成效，城乡爱国卫生、医疗服务、疾病控制工作进一步加强，“爱婴市”、农村初保和消灭丝虫病等通过省评审达标并得到巩固，市疾控中心、卫监所、慢病站相继建成。抗击“非典”工作卓有成效，我市成为零病例地区。全民健身计划得到落实，竞技体育取得新成绩。赈灾救济、优抚安置、社会福利、老区建设等工作得到加强，军政军民关系更加密切。民族、宗教、外事、侨务、旅游、统计、物价、气象、防震减灾、人民防空等工作取得新进步。妇幼、老龄、残疾人事业进一步发展。

七、民主法制建设得到加强

坚持和完善向人大及其常委会报告工作、向政协通报情况的制度，自觉接受监督，重视工会、共青团、妇联等群众团体的意见和建议，民主渠道进一步拓宽。五年来，共办理人大代表议案及建议203件、政协提案及建议323件。依法理顺农村基层管理体制，完成村民委员会换届选举工作，实行村民自治。城市基层管理体制改革、社区建设有序推进。重视人民群众来信来访工作。普法教育、法律服务工作得到加强。政务公开、村务公开、厂务公开普遍推行。审计和经济监督工作进一步加强。依法行政稳步推进，行政执法责任制和错案（过错）责任追究制进一步落实。行风评议活动扎实有效。廉政建设和反腐败斗争深入开展，抓好领导干部廉洁自律、查处大案要案、纠正部门和行业不正之风等工作取得成效。

八、人民生活水平进一步提高

2002年，全市城镇居民人均可支配收入6929元，农民人均纯收入3713元，年均分别增长5.9%和2.6%。城乡居民储蓄存款余额241.25亿元，年均增长12.7%。就业和再就业工作力度加大，城镇登记失业率控制在2.5%左右。养老、失业、医疗保险覆盖面不断扩大，国有企业下岗职工基本生活费和企业离退休人员养老金按时足额发放，城乡居（村）民最低生活保障制度逐步健全。农村困难家庭子女教育扶助政策得到落实。长期困扰揭东县枫江沿岸五镇25万群众和普宁市地氟区部分群众“饮水难”问题得到较好解决。扶贫开发和“两大会战”取得成效，脱贫奔康步伐加快。全市人民生活总体上达到小康水平。

各位代表！过去的五年，是不平凡的五年，成绩来之不易。这是省委、省政府和中共揭阳市委正确领导的结果，是各级人民代表大会及其常务委员会和人民政协监督支持的结果，是全市人民和衷共济、奋力拼搏和历届政府辛勤工作、艰苦创业的结果。在此，我代表市人民政府，向全市人民，向各民主党派、人民团体、工商联和各界人士，向中央和省驻揭单位，向人民解放军驻揭部队、武警官兵、公安干警表示崇高的敬意！向关心支持我市现代化建设的港澳台同胞、海外侨胞、海内外乡亲和友好人士表示衷心的感谢！

各位代表，五年来的工作实践，既奠定了坚实基础，又积累了宝贵经验。我们的主要体会是：必须坚持以邓小平理论和“三个代表”重要思想为指导，解放思想，实事求是，与时俱进，开拓创新，不断推动经济建设和社会各项事业的新发展；必须坚持以经济建设为中心，把上级要求与我市实际紧密结合，端正经济发展指导思想，创造性地开展工作，走有揭阳特色的发展路子；必须坚持把发展作为第一要务，放手发展民营经济，充分利用国际国内两个市场、两种资源，着力提高经济增长的质量和效益；必须坚持“稳中求快，强化管理”的工作方针，正确处理好经济建设和人口、资源、环境保护的关系，维护社会稳定，实现经济社会全面进步；必须坚持全心全意为人民服务的宗旨，执政为民，努力为群众办好事办实事，不断提高人民生活水平；必须坚持“两手抓，两手都要硬”的方针，加强思想道德文化建设，大力弘扬“聚三江人心，创时代伟业”的揭阳精神，促进物质文明、政治文明、精神文明协调发展。

五年来我们虽然取得了很大成绩，但也必须清醒地看到，在我市的发展中仍然存在许多困难和问题。主要是：经济结构不够合理，民营经济整体素质不高，工业化、城镇化和农业产业化水平较低；区域发展不平衡，县域经济实力不强，镇村集体经济比较困难；投资环境和创业环境还不尽人意，对外开放水平有待提高；城乡环境保护任务繁重，“脏乱差”和水污染问题仍然突出；社会治安综合治理和市场经济秩序整治难度较大，信用建设有待加强；社会保障体系仍较脆弱，就业和再就业压力较大，一些群众生活比较困难；财税基础比较薄弱，社会事业和市政建设欠帐较多；政府职能转变、机关作风建设和廉政建设还存在一些薄弱环节。对这些困难和问题，我们要高度重视，采取措施认真解决。

今后五年奋斗目标和主要措施

各位代表！未来五年，是我市加快改革开放、全面建设小康社会的关键时期。放眼世界，经济全球化迅猛发展，科技革命日新月异，市场竞争更趋激烈。党的十六大胜利召开，吹响了中华民族伟大复兴的号角，全国人民锐气昂扬，奋勇争先，现代化建设呈现百舸争流、千帆竞发之势。在改革发展的关键时刻，中共中央政治局委员、省委书记张德江同志考察粤东并发表重要讲话，为我们今后的发展提供了强大的思想武器和精神动力。刚刚闭幕的市第三次党代会，描绘了发展蓝图，确定了奋斗目标，指明了前进方向。面对新的机遇和挑战，我们要实现新的腾飞，必须紧紧抓住新世纪头二十年的重要战略机遇期，充分把握我国加入世贸组织、新一轮国际产业和国际资本向中国转移的历史机遇，充分利用省委省政府加大支持东西两翼发展的有利条件，充分借助我市交通便利、商贸活跃、民资民力和侨资侨力丰富的优势，充分发挥我市业已形成的物质技术基础和发展后劲，狠抓发展第一要务，强化忧患意识、责任意识和赶超意识，敢于冲破旧框框，勇于探索新路子，善于创造新优势，以大开放促大开发，以大开发促大发展，全面掀起新一轮建设热潮，实现经济社会的跨越式发展。

今后五年的指导思想：高举邓小平理论伟大旗帜，以“三个代表”重要思想为指导，全面贯彻党的十六大、胡锦涛总书记视察广东重要讲话精神，按照省委、省政府的战略部署和市第三次党代会的总体要求，以全面建设小康社会为总目标总任务统揽全局，积极实施“工业立市、科教兴市、外向带动、可持续发展和区域协调发展”五大战略，继续坚持“稳中求快，强化管理”工作方针，以工业化为龙头，以城镇化为载体，以外源型经济和内源型经济发展为动力，以大力发展民营中小型生产性轻工类企业为主攻方向，加快建设发达的轻工业基地，实现城乡经济整体推进，“三个文明”协调发展，努力开创我市改革开放和现代化建设的新局面。

今后五年的发展思路：坚定不移地推进工业化，以提高产业竞争力为核心，以工业园区为载体，以项目引进为突破口，大力发展民营中小型生产性轻工类企业，走新型工业化道路；坚定不移地推进城镇化，以市区为重点，以更加适宜创业发展和生活居住为目标，按照工业进园、住宅进区、农民进城的发展方向，调整完善规划，优化资源配置，加快建设步伐，打破城乡二元结构，形成以城带乡、以乡促城、城乡互动、共同繁荣的城乡一体化格局；坚定不移地推进农业产业化，以效益为中心，以市场为导向，以基地为依托，以龙头企业为骨干，发展现代农业，促进农村经济结构由农业主导型向工业主导型转变；坚定不移地推进信息化，完善信息平台，发挥网络优势，推进信息技术在各个领域的应用，提高经济发展和社会管理的信息化水平。

今后五年的奋斗目标：

综合实力要有新提高。国内生产总值以年均11%以上的速度增长，地方财政一般预算收入年均增长12%。经济结构进一步优化，三次产业构成比例更为合理。城乡面貌有较大改观，交通网、能源网、信息网、水利网基本形成，全社会固定资产投资年均增长14.5%，城镇化水平达50%以上。全市基本达到比较宽裕的小康水平。

民营经济要有新突破。发展环境更加优化，工业园区集聚效应更加显著，民营企业体制创新、科技创新、管理创新力度加大，民营经济发展规模明显扩大，整体素质明显提高，产业竞争力进一步增强。

县域经济要有新发展。区域资源优势得到充分发挥，特色经济、园区经济、劳务经济日益壮大，县、镇、村三级经济实力进一步增强。地区生产力布局更趋合理，区域经济协调发展。

改革开放要有新格局。各项改革不断深化，社会主义市场经济体制进一步完善，行为规范、运转协调、公正透明、廉洁高效的行政管理体制基本形成。对外开放向广度和深度拓展，管理体制与国际规则接轨加快，外源型经济快速增长，海纳百川、兼容并蓄的大开放格局逐步形成。

文化建设要有新进步。精神文明建设进一步加强，文化设施建设有较大进展，文化产业发展步伐加快，文化对经济的渗透力和影响力日益增强，文明法治环境逐步完善，市民文化素质和城乡文明程度进一步提高。

人民生活要有新水平。就业渠道不断拓宽，社会保障体系日益健全，教育、卫生、体育等事业进一步满足人民群众的需求，人与自然和谐发展，城乡人民生活水平和质量显著提高。城镇登记失业率控制在2.5%左右，城镇居民人均可支配收入年均增长6%，农民人均纯收入年均增长3.5%。

今后五年，我们要在新一轮竞争中赢得主动和优势，实现新的奋斗目标，必须集中精力抓大事，认认真真办实事，扎扎实实促发展。

一、大力发展民营经济，提高工业化水平

加大扶持发展力度。毫不动摇地鼓励、支持和引导非公有制经济发展，把发展民营经济作为一个大战略来抓，按照政治平等、政策公平、法律保障、放手发展的方针，优化政策、政务、法制、市场和舆论环境。放宽市场准入条件，凡符合国家法律法规、产业政策规定以及加入世贸组织后对内对外开放的行业和投资领域，都对民营企业开放。全力支持、发展壮大民营工业。加强民营科技园区和民营工业重点镇建设，重点支持科技型、外向型、从事农产品加工型和吸纳下岗人员就业型民营企业的发展。加大扶持力度，帮助解决发展用地、资金筹措等问题。设立市中小企业和民营企业管理机构，加强引导和服务。发挥商会和行业协会的作用。依法保护民营企业的合法财产和权益，努力为民营企业创业人员营造“社会上有地位、政治上有荣誉、经济上有实利”的良好发展氛围。

提高工业产业竞争力。利用高新技术尤其是运用信息技术改造提升传统产业，加强对纺织服装、食品饮料、五金机械、塑胶制品等传统优势产业的技术改造，促进传统产业的分化、替代、重组和整合，增强传统产业的竞争力。把发展高新技术产业作为第一增长点，大力培育和扶持具有一定基础和优势的光机电一体化、新材料、电子信息、医药化工等新兴产业。引导民营企业进行制度创新，加快公司制、股份制改造，建立现代企业制度。支持民营企业以资本为纽带，以市场为导向，组建跨地区、跨行业、跨所有制的企业集团。鼓励和支持有条件的民营企业上市，提高资本运营水平。实施名牌战略，发挥著名商标、知名商标和名牌产品的示范作用，培育和壮大一批在国内以至国际知名的品牌、商标和企业。推进国有企业和国有资产管理体制改革，进一步探索公有制经济的多种有效实现形式。

做大做强园区经济。大办工业园区，提升工业园区规划建设管理水平，营造“政策洼地”和“服务高地”，加速集聚工业发展要素，重点加快揭阳高新技术开发区、仙梅民营科技园、普宁科技工业园、揭西对外加工区和１２个对外工业园区的建设。抓紧建设揭阳（惠来）沿海经济开发试验区。依托地方特色产业，培育特色工业园区。创造条件，建设中心镇工业园区，为小城镇、行政村发展工业提供载体。

全方位招商引资。发展壮大园区经济关键在于招商引资，招商引资突破口在于项目引进。要推进观念更新和体制创新，建立招商引资责任制，实施招商引资奖励措施。提高招商水平，采取园区招商、规划招商、重点招商、网上招商、产业招商等多种形式招商，在重大项目的引进上寻求新的突破。积极承接国内先进地区特别是“珠三角”的产业转移，参与各种形式的区域经济合作。进一步发挥侨乡优势，以侨引侨，以侨引外，吸引更多的实业家和大企业到我市投资创业。要创造条件，千方百计吸引海内外的项目、资金、技术、人才参与我市的现代化建设。

二、统筹城乡发展，加快城镇化进程

加强基础设施建设。深化投融资体制改革，简化审批程序，对社会投资基建项目实行登记备案制度。推进投资主体多元化，继续加快交通网、能源网、信息网、水利网建设，重点抓好惠来电厂、潮汕民用机场、汕揭梅高速公路揭阳路段、揭潮高速公路、揭阳500千伏输变电站、市区防洪工程、莲花大道等重点项目的立项、建设工作。创造条件，争取更多项目纳入国家和省的计划“笼子”。

强化市区龙头带动作用。围绕把市区建成100万人口大城市的发展目标，高起点规划，高标准建设，高效能管理。树立“经营城市”理念，进一步加强政府对土地市场的调控管理。推进公用事业改革，积极探索公用事业社会化、市场化发展的新路子。按照“谁投资、谁经营、谁受益”的原则，全面放开城市经营性项目建设市场，鼓励各种所有制经济投资建设。发挥城市管理相对集中行政处罚权的作用，加强市区环境综合整治和“两河四岸”的开发建设。重视文化规划建设，提升城市文化品位。按照“统一规划，分步实施，重点突破，整体推进”的原则，把市区建设成为全市的政治、经济、文化、科技中心。

促进城乡联动发展。围绕把普宁市区建成50万人口、各县县城建成20万人口的中等城市，把其他13个中心镇建成5万以上人口城镇的发展目标，调整完善规划，加快建设步伐。积极推进小城镇和行政村的规划建设。加强城镇基础设施、公共服务设施、信息网络设施的建设，努力建设一批基础产业好、服务功能全、生态环境优、带动能力强的中心城镇。推进城镇户籍制度改革，打破不利于城镇化的体制和政策障碍，引导农村人口向城镇集中，乡镇工业向工业小区集中，走一条具有揭阳特色的城镇化道路。

三、推进农业产业化经营，全面繁荣农村经济

提高农业综合效益。以市场化思维办农业，推进农业产业化经营。巩固提高6大农业支柱产业和12个农业商品基地建设水平，加快建设农业现代化示范区和标准化示范园区。加大对15家省级、市级农业龙头企业扶持力度。加强农村市场体系建设，鼓励发展各种所有制形式的农产品加工企业和流通企业。抓好农业标准化工作，加强农产品质量安全监督。大力发展无公害农产品，积极发展绿色食品和有机食品。

加强农业基础建设。加大对农业防洪、灌溉、农田基本建设等方面的投入，重点做好市区防洪工程、惠来见龙海堤、凤山港海堤、揭西龙潭联围的建设以及石榴潭水库、普宁汤坑水库等工程的除险加固。搞好供水规划，做好引韩工程的有关工作。加大农村环境卫生治理力度，改善农村生产生活条件。

深化农村各项改革。认真贯彻“多予、少取、放活”的方针，千方百计增加农民收入。积极稳妥推进农村税费改革，切实减轻农民负担。全面落实农村土地承包政策，加强农村集体土地管理，引导和规范农户承包土地经营权的有序流转。积极推进供销社体制改革，抓好社产重组。继续搞好扶贫开发工作，加快老区、山区和贫困地区脱贫奔康步伐。

四、努力开拓市场，扩大消费需求

全方位开拓国内市场。加强商品市场建设，大力提升批发市场、专业市场和农副产品市场发展水平。积极参加省举办的各类展销会，办好我市的展销会、洽谈会，发展会展经济。拓宽消费领域，培育消费热点，促进连锁经营、现代物流、网上购物等新型业态的成长，大力发展旅游业、物业管理、社区服务、中介服务等行业。培育发展金融、技术、信息、劳动力、房地产等要素市场，完善市场体系。

多渠道拓展国际市场。应对经济全球化和我国加入世贸组织的新形势，主动参与国际竞争与合作，在更大范围、更宽领域、更高层次上扩大对外开放，做大做强外源型经济。扩大一般贸易出口，提高加工贸易水平，发展服务贸易。大力推行国际先进标准，不断提高出口商品质量。继续抓好纺织服装、鞋类、机电产品和不锈钢制品等大宗商品和名牌产品的出口。用足用好鼓励出口优惠政策，扩大民营企业出口。推进出口市场多元化，积极巩固港澳、东南亚、欧美日等传统市场，大力开拓中东、非洲、拉美、俄罗斯等新市场。鼓励有条件的企业“走出去”，与国际市场、技术和资本对接，积极参与国际竞争。加强协调服务，营造良好的通关环境。

五、加强区域协调，壮大县域经济

发展壮大工业经济。围绕农民增收、工业增效、财政增长、后劲增强的目标，坚持以工业化为核心，工业化、城镇化、农业产业化“三化”并举；坚持以开放为突破口，以开放促招商引资，促外引内联，促改革发展；坚持以工业园区为载体，千方百计加快项目建设。要突出重点产业，主攻民营经济，培育特色经济，壮大园区经济，发展配套经济，提升劳务经济。

促进区域协调发展。加强分类指导，发挥比较优势，形成基地化、专业化生产格局。市区要依托城市功能，提高工业园区发展水平，加大招商引资力度，推进城郊型经济发展，不断壮大经济实力，发挥辐射带动作用。普宁市要打响“青梅之乡”、“蕉柑之乡”、“青榄之乡”的品牌，发挥商贸活跃和服装、纺织、医药等产业的优势，依托衬衣节和中药材交易会，把普宁建设成为“富有活力的轻工名城和商贸名城”。揭东县要提升笋、果、菜等“三高”农业发展水平，发挥揭东经济开发区的综合效应，做大做强民营工业和园区经济，力争建成区域性的新兴制造业基地。揭西县要发挥政策优势和资源优势，发挥生态示范县品牌效应，加快工业园区建设，推动生态农业和生态旅游业的发展，提高工业化水平。惠来县要发挥临海优势，推进海洋、山地资源综合开发，加快工业化进程，力争在项目引进上获得重大突破。普宁华侨管理区和大南山华侨管理区要用足用好政策，做活“侨”字文章，壮大经济实力。

六、优化发展环境，增强综合竞争力

优化市场环境。以提高发展竞争力为核心，努力建设依法办事、诚实守信、文明服务、清洁卫生、安全有序的发展环境。继续整顿和规范市场经济秩序，深入开展专项整治，严厉打击偷税骗税、逃废债务、制假贩假、走私贩私、非法传销等违法犯罪活动。进一步落实安全生产责任制，强化城乡公共安全管理。依法治税理财，强化审计监督，加快公共财政体制建设。以提高政府公信力为核心，推进社会信用体系建设，逐步建立企业和个人信用制度，打造“信用揭阳”新形象。

优化治安环境。坚持“重典治乱”，加强社会治安综合治理，严厉打击各种严重刑事犯罪活动和黑恶势力，加大禁毒禁赌力度，坚决打击“法轮功”邪教组织破坏活动。加强基层治安基础建设，健全群防群治网络，完善打、防、控一体化的社会治安长效机制。坚持从严治警，建设高素质的政法队伍。重视群众来信来访，及时化解和妥善处理人民内部矛盾，维护社会稳定。

优化生态环境。强化人口、资源、环境管理，全面贯彻执行国家、省人口与计划生育法律法规，强化一把手亲自抓、负总责和“一票否决权”制度。强化人口与计划生育综合治理力度，抓好计生工作重心下移，加强基层基础建设，依法实行村（居）民自治。完善殡葬设施，巩固殡葬改革成果。以保护水资源为重点，强化榕江、练江、龙江、枫江等水域的保护和治理，切实治理工业污染、大气污染和生产生活噪音。以市区为重点，加快污水处理厂和垃圾处理场的建设，加强城镇公厕等公共卫生设施的配套建设。搞好“三江”流域生态公益林建设，加强森林公园、风景名胜区、自然保护区、生态示范区的建设和管理，依法保护土地、海洋、森林等资源。

七、优先发展教育科技事业，提高创新发展能力

大力发展教育事业。落实农村基础教育“以县为主”的管理体制，巩固提高普及九年义务教育，增加教育投入，优化资源配置。积极稳妥调整中小学布局，推进中小学信息技术教育和英语教育。加强学前教育。大力发展高中教育，建设示范性普通高中，重点解决高中学位不足的问题。加快建设揭阳市第一中学新校区和市第二实验小学。加快职业技术教育发展，继续办好揭阳职业技术学院。加强教师队伍建设，提高教育质量。发展继续教育，构建终身教育体系和学习型社会。运用市场机制促进社会力量办学。

推进科技改革和创新。继续深化科技体制改革，建立和完善以市场为导向、企业为主体、产学研相结合的技术创新体系，加速科技成果的转化和产业化。以民营企业为主体，引导和支持重点行业、大企业建立各类技术研究开发机构，创立自主知识产权，增强科技创新能力。积极做好高新技术企业和民营科技企业申报工作。加大科技兴农力度，办好农科示范基地，大力推广良种、良法。加强科普工作。依法保护知识产权。

加快国民经济信息化进程。重视发展信息产业，推动信息技术在各个领域的应用。发挥揭阳信息网络平台的作用，加快建设大容量、高速率的信息主干网。推动政务、企业、社会等领域信息资源的开发和利用，促进电子政务、电子商务发展。加强山区和农村信息化建设，逐步实现“网络到镇、信息进村”，建立全社会信息网络体系。

充分发挥人才效应。认真贯彻尊重劳动、尊重知识、尊重人才、尊重创造的方针，重视对人才的培养、提高、使用和管理，加快行政管理队伍、企业家队伍、经营管理队伍、科技人才队伍、熟练产业工人队伍和农村实用人才队伍的建设。建立富有生机活力的人才激励机制，为各类人才施展才华和干事创业营造良好环境。

八、拓宽就业渠道，完善社会保障体系

重视就业和再就业工作。坚持贯彻劳动者自主择业、市场调节就业、政府促进就业的方针，大力发展非公有制经济、第三产业、劳动密集型产业、社区服务业，增加就业岗位。引导人们转变就业观念，落实扶持政策，改善创业环境，支持失业下岗人员自谋职业、自主创业。加强就业服务，建立健全就业援助制度。依法加强劳动用工管理，保障劳动者合法权益。

完善社会保障体系。依法扩大社会保险覆盖面，完善城镇职工养老、医疗、失业保险和城乡居民最低生活保障制度，确保国有企业下岗职工基本生活费和离退休人员养老金按时足额发放。强化基金征缴，增强社保基金的保障能力。认真落实农村困难家庭子女免收义务教育阶段书杂费和符合条件免费入读技工学校等政策，重视解决弱势群体“四难”和部分地区群众饮水难等问题。完善社会救助网络，确保城乡群众无饥寒。关心和支持妇女、儿童、老龄和残疾人事业。

九、加强精神文明建设，协调发展社会事业

深入开展精神文明创建活动。坚持用邓小平理论和“三个代表”重要思想武装干部群众。大力弘扬民族精神、“抗非”精神和“聚三江人心，创时代伟业”的揭阳精神，最大限度调动人民群众干事创业的积极性。认真贯彻公民道德实施纲要，加强社会公德、职业道德和家庭美德教育。深入开展文明城市、文明村镇、文明行业等群众性精神文明创建活动，提高公民文化素质和城市文明程度。开展“除陋习，树新风”活动，移风易俗，倡导科学、健康、文明的生活方式。

全面推进先进文化建设。推进文化体制改革，发展文化事业和文化产业，促进文化与经济的有机融合。充分发挥历史文化名城优势，高度重视传统文化的改造，繁荣社区文化、企业文化、校园文化，与时俱进发展潮汕文化。积极筹建市广播电视中心、图书馆、博物馆、群艺馆、档案馆新馆等文化设施。做好文史档案工作，认真编修《揭阳市志》。大力发展新闻出版、广播电视、文学艺术、社会科学等事业。

加快发展卫生、体育等事业。深化医疗卫生体制改革，提高医疗卫生服务水平。健全公共卫生突发事件应急机制和疾病预防控制体系，提高对突发性公共卫生事件的快速应急处理能力。深入开展爱国卫生运动，做好卫生保健知识的宣传普及工作。进一步加强农村医疗卫生工作，建立农村新型合作医疗制度。规范药品经营、医疗市场秩序，保障群众医疗用药安全有效。努力发展体育事业，推动全民健身活动广泛开展。加快建设市体育馆和群众性休闲健身娱乐场所。抓好全民国防教育，做好双拥共建和优抚安置工作。提高统计服务水平。做好消防、气象、外事侨务、人民防空、防震减灾等工作。依法加强宗教事务管理。

十、加强民主法制建设，塑造政府良好形象

推进民主法制进程，提高依法行政水平。坚持向人大及其常委会报告工作、向人民政协通报情况的制度，自觉接受监督。认真办好人大代表议案、建议和政协提案。发挥民主党派、工商联、无党派人士参政议政作用和工会、共青团、妇联等群众团体的桥梁纽带作用，多方听取人民群众的意见和要求。健全基层自治组织，完善村民自治和居民自治，健全村务公开和厂务公开制度。认真抓好“四五”普法教育，提高全民法律素质，特别是公职人员的法制意识和依法行政能力。建立和完善了解民情、反映民意、集中民智、珍惜民力的决策机制，建立健全社会公示和专家咨询制度，推进决策科学化民主化。加强地方规范性文件立规工作，提高立规质量，强化执法监督，健全行政执法责任制和错案（过错）责任追究制，推进依法行政。

转变政府职能，加强作风建设。推进政府管理创新，改革行政管理体制，简化行政审批程序，提高行政效能。大力推行政务公开，实行首问责任制和服务承诺制，增强政府工作透明度，接受社会监督。始终牢记“两个务必”，努力做到“五个力戒”，强化公仆意识，时刻关注人民群众的冷暖安危，积极解决社会反映强烈的热点难点问题，竭力为老百姓办好事办实事。

牢记宗旨，干净干事。坚持为民执政，依法行政，廉洁从政。深入开展反腐败斗争，抓好领导干部廉洁自律，落实廉政建设责任制，纠正部门和行业不正之风，加大从源头上预防和治理腐败的力度。坚决查处大案要案，严惩腐败分子。切实加强公务员队伍建设，提高公务员队伍素质，努力做到权为民所用、情为民所系、利为民所谋，扎扎实实工作，干干净净干事，树立“廉洁、勤政、务实、高效”的政府形象。

全力以赴完成今年工作任务

各位代表，2003年，是落实本届政府任期目标的第一年，做好今年的工作，至关重要。今年我市经济和社会发展的主要预期目标是：国内生产总值增长11%左右；工业总产值增长13.5%；农业总产值增长5.5%；全社会固定资产投资增长14%；全社会消费品零售总额增长13%；外贸出口总额增长30%；实际利用外资增长15%；地方财政一般预算收入增长11%；人口自然增长率控制在9‰以内；城镇登记失业率控制在3%左右。为确保完成今年各项目标任务，我们要突出重点，只争朝夕，着力抓好以下几项工作：

一、抓好工业生产。引导企业适应市场变化，及时调整产品结构，扩大生产能力，组织适销对路产品开拓市场，提高产品市场占有率。加快技改和创新步伐，重点抓好康美药业、巨轮模具、运通集团、榕泰实业公司等27个列入国家、省技改导向计划项目和技术创新项目的研发、实施工作，抓好41个市技改导向计划项目和22个技改结转项目的实施工作。

二、抓好农业和农村工作。切实抓好夏收、夏种和晚造生产。做好防汛抗灾工作，确保安全渡汛。加紧落实省九届人大关于小型水库除险加固、移民、山区县电排电灌改造的议案。全面铺开、积极稳妥推进农村税费改革，切实减轻农民负担。做好计划生育工作，加大计生村民自治和司法支持力度，确保完成全年人口计划和计划生育各项管理任务。加快建立和完善农村新型合作医疗和农民最低生活保障制度。开展征用农民集体土地和土地征用款专项执法督察工作。

三、抓好固定资产投资。千方百计筹集建设资金，积极争取国债资金、银行资金和专项资金，加大利用外资力度，促进民间投资，加快年初确定的10项重点工程57个项目的建设。抓紧抓好市区临江南路、黄岐山大道绿化配套等项目建设。积极筹备召开市区工作会议，促进市区加快发展。

四、抓好民营经济发展。认真贯彻全市民营经济工作会议精神，全面落实市委、市政府《关于加快民营经济发展的实施意见》。进一步细化措施，强化服务，用足用好各项扶持政策，帮助民营企业解决实际困难，扎扎实实为民营企业发展营造良好的环境。

五、抓好商贸工作。积极参加省举办的各类招商洽谈会，认真筹办市在省外举办的经贸洽谈暨名优产品展销会。落实国家和省的有关优惠政策，帮助受“非典”影响较大的行业和企业克服困难，加快发展。加大招商引资力度，加强和改善服务，鼓励现有外商投资企业增资扩产。用足用好政策，加快出口退税进度。做好老客户、重点产品、出口大户的工作，千方百计扩大外贸出口。加强税务、金融、海关、外经贸、检验检疫等部门的协调配合，营造良好的外贸环境。

六、抓好财税工作。加强税收征管，着力抓好重点税源、重点税种的征管和稽查工作。大力清收欠税，做到应收尽收。加快公共财政体制建设，强化预算管理，努力增收节支。加强对非税收入的征管，强化“收支两条线”管理，推进部门预算改革。从严从紧控制财政支出，保吃饭、保稳定、保重点。强化和改进预算外资金监管，加强审计监督，严肃财经纪律。全面实行行政事业性收费公示制度。

七、抓好综合整治工作。继续深入整顿和规范市场经济秩序，开展专项整治行动，重点打击制贩假烟假币等违法犯罪行为，进一步巩固打击非法传销成果，防止反弹。强化安全生产监督和管理，坚决消除事故隐患。加强社会治安综合治理，加大对“两抢一盗”、吸毒贩毒等违法犯罪活动的打击力度，严密防范、坚决打击“法轮功”邪教组织破坏活动，全力维护社会稳定。

八、抓好社会各项事业的发展。抓紧建设揭阳市第一中学新校区。积极筹建市第二实验小学。严防死守，巩固成果，抓好“非典”等传染病的防治工作。广泛开展爱国卫生运动和全民健身运动，提高群众健康水平。认真解决部分地区群众饮水难问题，加快普宁市东部地区和揭西县棉湖片区供水工程建设，加快揭东县新亨镇、锡场镇和月城镇供水管网改造步伐。加强环境保护，搞好城乡净化、绿化、美化工作，营造良好的创业环境和生活环境。

各位代表！回顾过去，成绩令人鼓舞；展望未来，目标催人奋进！让我们紧密团结在以胡锦涛同志为总书记的党中央周围，高举邓小平理论伟大旗帜，努力实践“三个代表”重要思想，认真落实张德江同志考察粤东重要讲话和市第三次党代会精神，在市委的坚强领导下，抓住机遇，与时俱进，开拓创新，奋发进取，为全面建设小康社会、开创我市改革开放和现代化建设新局面而努力奋斗！
